# Associations Between Chronic Diseases and Neck Pain Characteristics: A Cross-Sectional Study

**DOI:** 10.3390/healthcare14193211

**Published:** 2026-09-29

**Authors:** Saud F. Alsubaie

**Affiliations:** Department of Health and Rehabilitation Sciences, College of Applied Medical Sciences, Prince Sattam bin Abdulaziz University, Alkharj 11942, Saudi Arabia; s.alsubaie@psau.edu.sa; Tel.: +966-540588079

**Keywords:** rehabilitation, geriatrics, musculoskeletal pain, gender differences, risk factors, comorbidity

## Abstract

**Highlights:**

**What are the main findings?**

- Participants with at least one chronic disease reported greater neck pain intensity and longer pain duration than those without chronic disease.
- After adjustment for age, sex, and BMI, chronic disease status remained independently associated with both pain intensity and pain duration.

**What are the implications of the main findings?**

- Chronic disease status should be considered when assessing and managing adults with neck pain.
- The findings support a more comprehensive assessment of comorbidity and overall health status in people with neck pain.

**Abstract:**

**Background**: Neck pain is a common musculoskeletal condition that can adversely affect functional ability and quality of life. Although chronic diseases are thought to influence the burden of neck pain, evidence regarding their relationship with neck pain intensity and duration remains limited. **Aim**: To examine the association between chronic diseases and the intensity and duration of neck pain among adults. **Design**: Observational cross-sectional study. **Setting**: Adult population in Saudi Arabia. **Methods**: Participants aged 18–65 years completed a questionnaire assessing demographic characteristics, chronic disease status, and neck pain intensity and duration. Comparisons were conducted between participants with and without chronic disease in terms of pain intensity and duration. Multivariable linear regression analyses were used to examine the associations between chronic disease and neck pain intensity and duration, after adjustment for age, sex, and body mass index (BMI). **Results**: A total of 2036 participants (mean age 32.71 ± 11.28 years; 61.6% women) were included in the study. Overall, 323 participants (15.9%) reported at least one chronic disease. Participants with at least one chronic disease reported significantly greater neck pain intensity and longer pain duration than those without chronic disease (both *p* < 0.001). After adjustment for age, sex, and BMI, the presence of at least one chronic disease was associated with greater neck pain intensity (B = 0.532, 95% CI 0.255–0.808; *p* < 0.001) and longer pain duration (B = 0.369, 95% CI 0.155–0.583; *p* = 0.001). **Conclusions**: The presence of chronic disease was independently associated with greater neck pain intensity and longer pain duration after adjustment for age, sex, and BMI. These findings highlight the need for clinical assessments that take into consideration chronic disease status when assessing and managing individuals with neck pain.

## 1. Introduction

Neck pain is of the most common musculoskeletal disorders affecting people of all age groups worldwide. Neck pain is associated with limited neck movement and can impact quality of life and place a burden on the healthcare system [1,2]. This problem is expected to increase due to modern lifestyles that require increased use of computers in office work, smartphones, and other electronic devices. One study indicates that neck pain affected 203 million people worldwide in 2020, and that this number is expected to increase by 32.5% by 2050 to reach 269 million people globally [1]. According to a report from the World Health Organization, neck pain is a common musculoskeletal problem affecting more than 220 million people worldwide and is one of the leading contributors to global disability [3].

Neck pain can lead to impaired daily functioning, decline in overall quality of life, and reduced work performance, in addition to causing some days of absence from work. This indicates that this problem affects not only individuals, but also society [2]. Several demographic and biomechanical factors have been identified as potential risk factors for neck pain, including older age, female sex, obesity, prolonged sitting, repetitive neck movements, and poor posture [1,2,4,5].

Previous research has given limited attention to the relationship between chronic diseases and the development of musculoskeletal disorders, especially neck pain. Chronic diseases are defined as health conditions that last one year or more and generally require ongoing medical attention and may affect daily functioning [6]. In this study, chronic diseases were identified based on participants’ reports of medically diagnosed chronic conditions, including diabetes mellitus, hypertension, hypotension, osteoarthritis, lung disease, heart disease, dyslipidemia, kidney disease, thyroid disease, and cancer. However, most available studies have focused on the association between chronic diseases and lower back pain, highlighting the fact that chronic diseases in general contribute to greater pain severity, persistent pain, and poorer response to treatment [7,8]. Although some evidence has shown an association between multimorbidity and neck pain [9], the contribution of chronic diseases to neck pain, whether as potential risk factors or determinants of pain manifestation, has not been adequately investigated in the literature. Several factors may explain the relationship between chronic diseases and neck pain. Chronic diseases may lead to persistent inflammation, tissue changes, and reduced physical function, which may increase the risk of musculoskeletal pain or make pain last longer. In addition, psychological and social factors related to chronic diseases may affect how individuals experience and cope with pain. These factors may contribute to greater neck pain intensity and longer pain duration among individuals with chronic diseases.

Despite increasing interest in factors associated with musculoskeletal disorders, evidence on the influence of chronic diseases on neck pain characteristics, particularly pain intensity and duration, is still limited. In addition, it is unknown whether these relationships vary according to age and sex in community-based populations. A better understanding of these relationships may enhance strategies of risk reduction and support more comprehensive assessment and management of individuals with neck pain. Therefore, this study aimed to examine the association between common chronic diseases and neck pain intensity and duration among adults aged 18–65 years in Saudi Arabia.

## 2. Materials and Methods

### 2.1. Study Design and Participants

This cross-sectional study was designed to collect information from adults living in Saudi Arabia who had experienced neck pain within the last 12 months. A convenience sampling approach was used to recruit eligible participants from the community and outpatient physical therapy clinics in the Riyadh region. The questionnaire was administered electronically as a self-administered survey. Participants recruited from outpatient physical therapy clinics were provided with the study information and a survey link to complete the questionnaire independently. For community participants, the survey link was distributed through online communication channels. All participants reviewed the study information and provided electronic informed consent before completing the questionnaire. The inclusion criteria were adults aged 18–65 years who had experienced at least one incidence of neck pain within the previous 12 months. The exclusion criteria were a previous fracture, surgery, tumors, or neurological disorders in the cervical area. All potential participants were informed about the purpose and methods of the study. Those who agreed to participate in the study provided electronic informed consent prior to participation. Ethical approval was obtained from the Scientific Research Ethics Committee at Prince Sattam bin Abdulaziz University, Al-Kharj, Saudi Arabia (No. RHPT/026/001 on 5 January 2026).

### 2.2. Sample Size Calculation

The required minimum sample size was estimated using G*Power software (version 3.1.9.7) based on a multiple linear regression model. The calculation was performed using an F test for linear multiple regression (fixed model, R^2^ deviation from zero), assuming a medium effect size (f^2^ = 0.15), an alpha level of 0.05, a statistical power of 80%, and 13 predictors. The minimum required sample size was approximately 131 participants. However, a larger sample was recruited to provide adequate representation of participants with different chronic diseases and to support the primary and disease-specific analyses.

### 2.3. Outcome Measures

A new questionnaire was developed by the researcher specifically for this study based on the study objectives. The questions were designed to be simple and direct to obtain common demographic information and information about chronic disease and neck pain. Before data collection, the questionnaire was reviewed by two expert physical therapists who were not involved in the study, and their feedback was considered to improve the clarity and appropriateness of the questions. Participants were asked whether they had any medically diagnosed chronic disease. They could report more than one chronic disease if applicable. The chronic conditions reported by participants included diabetes mellitus, hypertension, hypotension, osteoarthritis, lung disease, heart disease, dyslipidemia, kidney disease, thyroid disease, and cancer. Additionally, participants reported the intensity of their average neck pain using a numerical rating scale from 0 (indicating no pain) to 10 (indicating worst possible pain). In addition, participants reported their neck pain duration by the number of cumulative days they experienced pain during the past 12 months. Neck pain was defined in a large epidemiological study as pain, tension, or stiffness localized to the cervical region, with possible radiation to the arm, lasting at least 24 h [10].

### 2.4. Statistical Analysis

Descriptive statistics were used to summarize demographic characteristics, neck pain characteristics, and chronic disease prevalence. Continuous variables were summarized using means and standard deviations or medians and interquartile ranges, as appropriate, whereas categorical variables were summarized using frequencies and percentages. The normality of the data of the continuous variables were tested using the Shapiro–Wilk test along with visual inspection. Comparisons were conducted between participants with and without at least one chronic disease. Pain intensity was compared using an independent-samples *t* test, whereas pain duration was compared using the Mann–Whitney U test because it showed a markedly right-skewed distribution.

Two primary multivariable linear regression models were fitted to examine whether the presence of at least one chronic disease was independently associated with neck pain intensity and pain duration after adjustment for age, sex, and body mass index. Pain intensity was treated as a continuous variable on the 0–10 numerical rating scale, and pain duration was recorded as the number of days and treated as a continuous variable. For the regression models, multicollinearity was assessed using tolerance and variance inflation factor (VIF), and residual plots were inspected to assess model assumptions. Because pain duration showed strong right-skewness, it was transformed using the natural logarithm before regression analysis to reduce skewness and improve the suitability of the outcome for linear regression. To assess the robustness of the primary regression findings, a supplementary bias-corrected and accelerated (BCa) bootstrap analysis based on 1000 resamples was performed. Secondary analyses examined each chronic disease separately in adjusted multivariable linear regression models controlling for age, sex, and BMI. All statistical analyses were two-sided, and statistical significance was set at *p* < 0.05. All statistical analyses were performed using IBM SPSS Statistics, version 25 (IBM Corp., Armonk, NY, USA).

## 3. Results

A total of 2036 participants were included in this cross-sectional study with a mean age of 32.71 ± 11.28 years (range, 18–65 years), and women represented 61.6% of the sample. The demographic and clinical characteristics of the study population are summarized in Table 1. Overall, 323 participants (15.9%) reported at least one chronic disease. The most commonly reported conditions were diabetes (5.1%), osteoarthritis (4.7%), and hypertension (4.5%). Chronic disease prevalence varied significantly across both sex and age groups, with higher rates observed among women and older adults (Table 1).

Participants with at least one chronic disease had significantly higher neck pain intensity (5.42 ± 2.37 vs. 4.60 ± 2.09; *p* < 0.001) and longer pain duration (median, 4 vs. 2 days; Mann–Whitney U = 199,977; *p* < 0.001) compared with participants without chronic disease (Table 2).

After adjustment for age, sex, and BMI, participants with at least one chronic disease had significantly greater neck pain intensity (B = 0.532, 95% CI 0.255 to 0.808; *p* < 0.001) and longer log-transformed pain duration (B = 0.369, 95% CI 0.155 to 0.583; *p* = 0.001) compared to those without chronic disease (Table 3).

Supplementary BCa bootstrap analyses with 1000 resamples confirmed the robustness of the primary findings. In the disease-specific adjusted models, osteoarthritis, hypotension, and thyroid disease were significantly associated with greater pain intensity after adjustment for age, sex, and BMI. Among these conditions, osteoarthritis showed the strongest association with pain intensity. For pain duration, osteoarthritis was the only individual chronic disease significantly associated with longer log-transformed pain duration (Table 4).

## 4. Discussion

In this study sample, 15.9% of participants reported at least one chronic disease. After controlling for age, sex, and BMI, chronic disease status remained independently associated with both greater neck pain intensity and longer pain duration. These findings suggest that comorbidity contributes to the clinical presentation of neck pain beyond the influence of demographic and anthropometric differences.

The results of this study showed that chronic disease status was independently associated with greater neck pain intensity and longer pain duration after adjustment for age, sex, and BMI, which is consistent with previous studies linking chronic conditions and multimorbidity to a higher burden of pain [8,11,12,13]. Several biological and psychological mechanisms may explain the association between chronic diseases and greater neck pain intensity and duration. Chronic diseases may be accompanied by persistent inflammation, tissue damage, impaired physical function, and other physiological changes that may increase susceptibility to musculoskeletal pain or contribute to its persistence. In addition, psychological and social factors associated with living with a chronic disease may influence pain perception and dealing with pain [14,15,16]. These possible mechanisms may explain why participants with chronic diseases reported greater pain intensity and longer pain duration even after adjustment for age, sex, and BMI. However, because of the cross-sectional design of the study, these mechanisms should be considered possible explanations rather than evidence of a causal relationship.

In the disease-specific analyses, osteoarthritis was the only chronic disease associated with both neck pain intensity and duration. The association between osteoarthritis and both outcomes is clinically probable, as osteoarthritis is a musculoskeletal condition that can be accompanied by joint-related pain and functional limitations. This finding is also consistent with previous research reporting an association between arthritis and greater pain burden in patients with lower back pain [8,17]. Thyroid disease was associated with greater pain intensity but not pain duration, which is in line with previous research reporting an association between thyroid disorders and pain [18,19]. Hypotension was also associated with greater pain intensity. One possible explanation is that the altered autonomic and baroreceptor-related regulation associated with low blood pressure may influence pain modulation [20]. The underlying mechanism of this association remains unclear, and further studies are needed to determine whether this finding can be replicated. However, this finding should be interpreted cautiously given the relatively small number of participants who reported hypotension. Because of the small number of participants with certain chronic diseases, each condition was examined in a separate adjusted model, and the analyses did not account for the presence of multiple chronic conditions within the same participant. The descriptive analyses showed clear differences in the distribution of chronic diseases across age groups. Among participants aged 50–65 years, hypertension was the most prevalent chronic condition (23.2%), followed by osteoarthritis (22.6%) and diabetes (21.4%). These findings indicate that cardiovascular, musculoskeletal, and metabolic disorders represent the most common chronic diseases in this population. Compared to other studies from other regions, our findings were similar, with high blood pressure consistently reported as the most common chronic disease among elderly people, although its rates vary between different studies [21,22,23,24,25]. Osteoarthritis and diabetes have also been frequently reported among older adults in numerous studies, although their prevalence varies across different studies. The prevalence of these chronic diseases is largely consistent with the findings of this study [21,22,23,24,25]. The observed differences in prevalence rates across studies may be attributed to several factors that can influence the reported prevalence, such as differences in the age groups included in those studies, sample characteristics, lifestyle behaviors, environmental exposures, and differences in the definition of chronic conditions. The study finding regarding the prevalence of chronic diseases among older adults is consistent with the findings of several previous studies. Furthermore, numerous studies have found that the likelihood of developing multiple types of chronic diseases increases with advancing age [8,25]. Many studies attribute the increased prevalence of chronic diseases with age due to exposure to some biological, physiological, and environmental factors over the lifespan. As individuals grow older, they become more exposed to risk factors and age-related physiological changes, which make them more susceptible to the onset and progression of chronic diseases. In addition, social and psychological factors such as low social support and attachment, high stress levels, lack of psychological support, and difficulty accessing healthcare can increase the likelihood of developing chronic diseases [24,25].

This study, along with many previous studies, indicated that women are generally more vulnerable to chronic diseases than men [8,22,24,26]. Regarding the types of chronic diseases most prevalent among genders, previous research reported that women are more likely to develop musculoskeletal disorders, such as osteoarthritis and other joint-related conditions, whereas men are more prone to conditions like diseases of the prostate [8,22,24,26]. Although this study found that women experienced pain for a longer period compared to men, it did not find a difference in pain intensity between women and men, contrary to many previous studies that indicated that both the intensity and duration of pain are usually higher in women [26,27,28,29,30,31,32]. Several studies attributed the differences between women and men in terms of how they perceive pain to the differences in biological and psychosocial characteristics [29,30,31,32]. Interestingly, some studies have shown that although men with chronic pain tend to report lower pain intensity compared to women, they often report a poorer quality of life. This suggests that men tend to underreport their pain severity even though it has an impact on their psychological and social life, leading to poorer quality of life despite reporting lower estimations of pain intensity [26].

The prevalence of chronic diseases increased with advancing age and differed between men and women. These findings contribute to the limited community-level epidemiological data available in Saudi Arabia and may help inform future public health planning and preventive strategies.

This study has some limitations that should be noted. First, this study used a cross-sectional design; therefore, it can only indicate associations between the study variables and cannot determine whether chronic diseases actually cause an increase in pain intensity or duration. Second, residual confounding cannot be excluded, as factors such as physical activity, occupational factors, socioeconomic status, smoking, and psychological factors were not assessed. In addition, the use of convenience sampling from both the community and outpatient physical therapy clinics may have introduced selection bias, as participants recruited from clinical settings may differ from those recruited from the community in terms of pain characteristics and chronic disease status. Additionally, chronic disease status was self-reported and was not verified using medical records, which may have introduced misclassification and recall bias. Another limitation is that the study did not measure the chronicity or severity of the chronic diseases or the medications used to manage them, which could have significantly impacted the pain characteristics of the study participants. Future longitudinal studies are warranted to better understand how changes in chronic disease status over time influence the characteristics of neck pain and other musculoskeletal pain conditions.

## 5. Conclusions

The presence of at least one chronic disease was independently associated with greater neck pain intensity and longer pain duration after adjustment for age, sex, and BMI. Among the individual chronic diseases examined, osteoarthritis showed the most consistent association with both outcomes. These findings highlight the importance of considering comorbidity and overall health status when assessing and managing adults with neck pain. Given the cross-sectional design of this study, the observed associations should not be interpreted as evidence of causality.

## Figures and Tables

**Table 1:** Demographic characteristics and prevalence of chronic diseases according to sex and age group.

Characteristic	Overall (*n* = 2036)	Female (*n* = 1254)	Male (*n* = 782)	*p*-Value	18–34 Years (*n* = 1192)	35–49 Years (*n* = 676)	50–65 Years (*n* = 168)	*p*-Value
Age (years), mean ± SD	32.71 ± 11.28	33.56 ± 10.96	31.36 ± 11.65	<0.001	24.41 ± 4.52	41.95 ± 4.34	54.46 ± 3.78	—
BMI (kg/m^2^), mean ± SD	26.65 ± 6.61	26.82 ± 6.83	26.37 ± 6.22	0.134	24.52 ± 5.88	29.22 ± 5.89	31.53 ± 7.98	<0.001
Any chronic disease, *n* (%)	323 (15.9)	236 (18.8)	87 (11.1)	<0.001	80 (6.7)	155 (22.9)	88 (52.4)	<0.001
Diabetes mellitus, *n* (%)	103 (5.1)	64 (5.1)	39 (5.0)	0.907	15 (1.3)	52 (7.7)	36 (21.4)	<0.001
Hypertension, *n* (%)	91 (4.5)	60 (4.8)	31 (4.0)	0.384	10 (0.8)	42 (6.2)	39 (23.2)	<0.001
Hypotension, *n* (%)	36 (1.8)	35 (2.8)	1 (0.1)	<0.001	11 (0.9)	19 (2.8)	6 (3.6)	0.002
Osteoarthritis, *n* (%)	95 (4.7)	79 (6.3)	16 (2.0)	<0.001	24 (2.0)	33 (4.9)	38 (22.6)	<0.001
Lung disease, *n* (%)	31 (1.5)	21 (1.7)	10 (1.3)	0.478	17 (1.4)	12 (1.8)	2 (1.2)	0.785
Heart disease, *n* (%)	14 (0.7)	9 (0.7)	5 (0.6)	0.835	1 (0.1)	9 (1.3)	4 (2.4)	<0.001
Dyslipidemia, *n* (%)	6 (0.3)	1 (0.1)	5 (0.6)	0.023	1 (0.1)	1 (0.1)	4 (2.4)	<0.001
Kidney disease, *n* (%)	11 (0.5)	8 (0.6)	3 (0.4)	0.446	9 (0.8)	1 (0.1)	1 (0.6)	0.227
Thyroid disease, *n* (%)	20 (1.0)	19 (1.5)	1 (0.1)	0.002	0 (0.0)	18 (2.7)	2 (1.2)	<0.001
Cancer, *n* (%)	0 (0.0)	0 (0.0)	0 (0.0)	—	0 (0.0)	0 (0.0)	0 (0.0)	—

Abbreviations: BMI, body mass index; SD, standard deviation; *n*, number of participants.

**Table 2:** Comparison of neck pain intensity and duration between participants with and without chronic disease.

Outcome	No Chronic Disease	Any Chronic Disease	*p*-Value	Effect Size
Pain intensity, mean ± SD	4.60 ± 2.09	5.42 ± 2.37	<0.001	d = 0.38
Pain duration, median (days)	2	4	<0.001	r = 0.16

Note: Pain intensity was compared using an independent-samples *t*-test, and pain duration was compared using the Mann–Whitney U test. Abbreviations: SD, standard deviation; d, Cohen’s d effect size; r, effect size calculated from the Mann–Whitney U test.

**Table 3:** Primary adjusted associations between any chronic disease and neck pain intensity and log-transformed pain duration.

Outcome	B	95% CI	*p*-Value
Pain intensity	0.532	0.255 to 0.808	<0.001
Ln-transformed pain duration	0.369	0.155 to 0.583	0.001

Abbreviations: B, unstandardized regression coefficient; CI, confidence interval.

**Table 4:** Adjusted associations between individual chronic diseases and neck pain intensity and log-transformed pain duration.

Chronic Disease	Pain Intensity B (95% CI)	*p*-Value	Ln-Pain Duration B (95% CI)	*p*-Value
Diabetes	0.085 (−0.354 to 0.524)	0.704	−0.240 (−0.582 to 0.101)	0.168
Osteoarthritis	0.631 (0.163 to 1.100)	0.008	0.983 (0.626 to 1.340)	<0.001
Hypertension	0.409 (−0.077 to 0.894)	0.099	0.160 (−0.217 to 0.537)	0.406
Hypotension	0.746 (0.029 to 1.464)	0.042	0.504 (−0.052 to 1.060)	0.076
Lung disease	−0.025 (−0.781 to 0.731)	0.948	0.441 (−0.154 to 1.036)	0.146
Thyroid disease	1.791 (0.848 to 2.733)	<0.001	0.554 (−0.178 to 1.287)	0.138
Heart disease	1.050 (−0.115 to 2.216)	0.077	0.171 (−0.733 to 1.074)	0.711
Dyslipidemia	−0.743 (−2.462 to 0.977)	0.397	−0.471 (−1.803 to 0.861)	0.488
Kidney disease	0.795 (−0.529 to 2.119)	0.239	0.521 (−0.504 to 1.547)	0.319

Abbreviations: B, unstandardized regression coefficient; CI, confidence interval.

## Data Availability

The data used in this study are available from the author upon reasonable request. The data are not publicly available due to privacy or ethical restrictions.
